# An Unusual Localization of a Pleomorphic Adenoma in the Rhinopharynx

**DOI:** 10.1155/2012/253186

**Published:** 2012-08-13

**Authors:** Fabio Pagella, Francesco Chu, Alessandro Pusateri, Elina Matti

**Affiliations:** Department of Otorhinolaryngology, University of Pavia and Foundation I.R.C.C.S. Policlinico San Matteo, 27100 Pavia, Italy

## Abstract

Pleomorphic adenoma is the most common benign tumor of the parotid glands. Rarely it may arise from minor salivary glands of the upper aerodigestive tract. A 57-year-old woman was admitted at our institution presenting with nasal obstruction. Endoscopic evaluation revealed a pedicled mass in the rhinopharynx. After radiological examination, we opted for a transnasal endoscopic-assisted excision of the mass under general anaesthesia. Histological evaluation deponed for pleomorphic adenoma with clear surgical margins. No endoscopic evidence of local recurrence has been shown after 48 months of followup. In the literature, few cases of pleomorphic adenoma arising in the rhinopharynx have been reported. The introduction of endoscopy, as shown by our experience, leads to important benefits in the identification, treatment, and followup of such rhinopharyngeal benign tumors.

## 1. Introduction

Salivary glands tumors can be classified into major and minor according to their site of origin. The first group includes tumors arising from the parotid, sublingual, and submandibular glands, while the second group includes neoplasms arising from the numerous minor salivary glands placed in the submucosa, along the upper aerodigestive tract. Pleomorphic adenoma, also known as “mixed tumor,” is a benign tumor affecting the parotid glands in 80% of cases. It represents 40–45% of all the parotid glands tumors [1]. Nasal localization is uncommon as shown by the large case series reported by Spiro et al. [2] and Compagno and Wong [3]. Moreover, in this group, even more uncommon are the tumors arising from the minor salivary glands of the rhinopharynx. In fact, only 6 adult case reports have been described in the literature [4–9]. Here we present a case of pleomorphic adenoma unusually arising in the rhinopharynx followed by a literature review in order to identify the major characteristics of such rare disease.

## 2. Case Presentation

**Figure 1:**
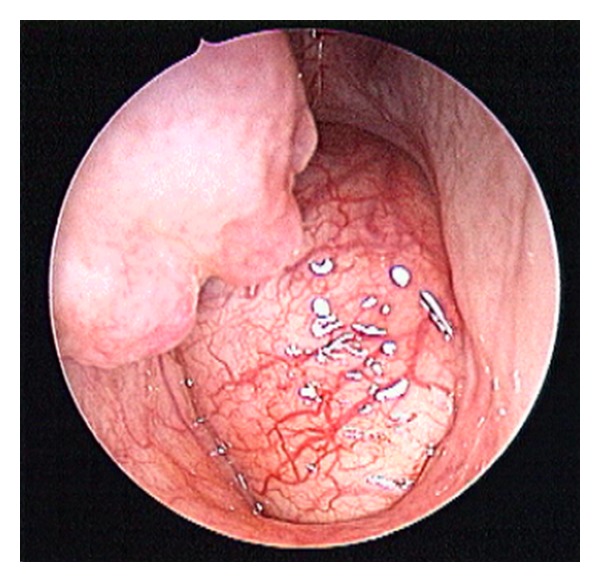
Endoscopic view of the right nasal fossa: the voluminous polypoid mass completely obstructing the choanae.

**Figure 2:**
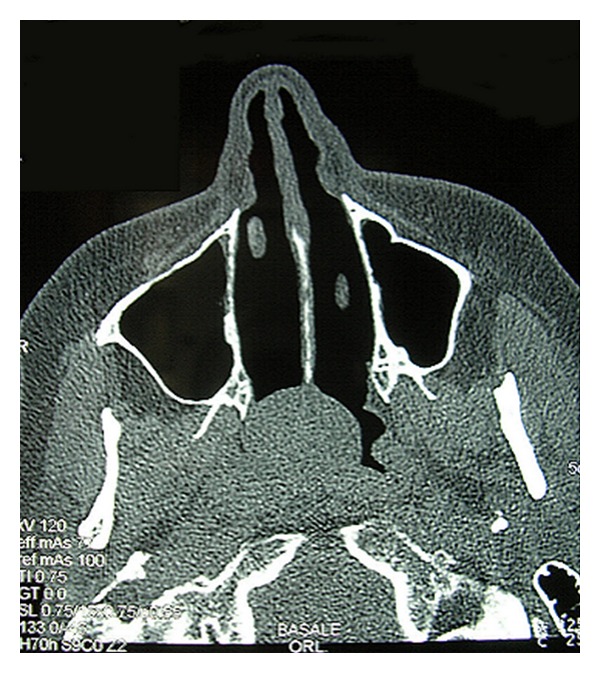
Axial CT scan: the polypoid mass occupying the rhinopharynx with no signs of bony framework erosion or infiltration.

A 57-year-old woman was referred to our department presenting with progressive nasal obstruction over the last months, not responsive to treatment with nasal decongestants. No other symptoms were reported. This patient underwent a nasal endoscopy which revealed the presence of a voluminous, pink-grey coloured polypoid mass pedicled in the rhinopharynx, not bleeding on touch, and obstructing the nasal choanae bilaterally (Figure 1). A computed tomography (CT) scan evidenced a mass occupying the rhinopharynx with no signs of bony framework erosion infiltration (Figure 2). Therefore, we opted for an excisional biopsy of the neoplasm by an endoscopic-guided endonasal approach.

The procedure was performed under general anaesthesia with the patient placed in anti-Trendelenburg position. Cottonoid pledgets, soaked in xylometazoline hydrochloride 0.1% solution, were positioned in the nasal cavity and left in place for about 10 minutes in order to achieve optimal decongestion and access to rhinopharynx. We identified the lesion's pedicle in the right Rosenmuller fossa and we removed it with cutting instruments. Assisted by powered shavers (XPS Xomed Powered System by Medtronic, Jacksonville, FL, USA), equipped with a 2.9 mm tricut blade, and straight-through suction irrigation, we completed the excision at the site of origin preserving the integrity of the Eustachian tube's ostium. The mass was transorally removed en bloc. Hemostasis was obtained with bipolar forceps. The patient was discharged the next day with medical therapy.

**Figure 3:**
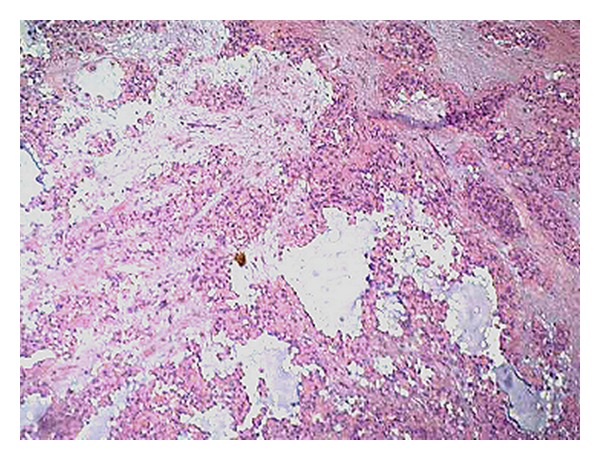
Admixture of polygonal and spindle-shaped epithelial elements in a background of a myxoid stroma. Original magnification (H-E, 20x).

Macroscopic examination revealed a grey, voluminous (2.8 × 1.8 × 1.8 cm), nodular mass with smooth surface, and a firm-elastic parenchyma consistence enveloped in a fibrous capsule. The microscopic pattern was composed by both epithelial and mesenchymal elements, multiple areas of metaplasia with myxoid and fibrous tissues deponing for the diagnosis of pleomorphic adenoma (Figure 3). Clear surgical margins were obtained and no additional surgical treatment was required.

**Figure 4:**
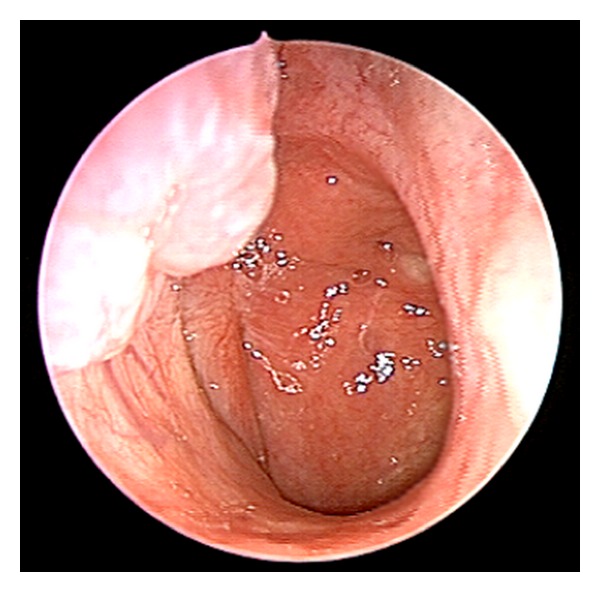
Endoscopic view of the right nasal fossa 12 months after-operation. No evidence of local recurrence.

After 48 months of followup, our patient is still asymptomatic and shows no endoscopic evidence of local recurrences (Figure 4).

## 3. Discussion

Pleomorphic adenoma, also known as “mixed tumor,” is a benign neoplasm usually arising from the salivary glands, both major and minor, with the first location more frequent than the latter [10]. It origins from the epithelial and myoepithelial cells of the intercalar ducts and its histologic pattern is characterized by the presence in its contest of different types of tissues (glandular, epithelial, myoepithelial, myxoid, fibrous, condral, and bony). Its high cellularity and solid growth can lead to misdiagnoses of more aggressive neoplasms [5]. For such reason, histologic examination of the entire specimen is advisable.

Its pathogenesis is not clear yet and the only risk factor clearly associated with the disease is represented by ionized radiation. As reported in the literature, the tumor shows an overall female preponderance and it affects the middle decades of life [10]. It represents the most common benign tumor of the parotid glands; its propensity to malignant transformation has been reported to vary between 1.9 and 23.3%; carcinoma ex pleomorphic adenoma occurs in 12% of all salivary malignancies [10–19].

After a literature review, we found only 6 previous adult cases of pleomorphic adenoma of the rhinopharynx [4–9]. The symptoms of this particular localization are frequently aspecific and potentially misinterpreting. In our case, the only symptom of the disease was a progressive nasal obstruction. In the case reported by Lee et al. [7], the neoplasm was responsible of a middle ear effusion with otalgia, tinnitus, fullness, and hearing loss without any obstructive manifestation. In our case, the lesion was mechanically occluding the ostium of the Eustachian tube but the tympanogram was an A type. Moreover, no fullness, pain, hypoacusia, or any other symptoms indicating a middle ear involvement were present at the moment of the diagnosis. Epistaxis and blood-stained discharge were referred in the case reported by Amilibia and in the case described by Lam et al. [5, 6]. In our case, even if the symptoms of this particular tumor localization were few and aspecific, nasal endoscopy led us to an early diagnosis of the rhinopharyngeal lesion. In the past years, the management of a nasopharyngeal lump could be quite difficult: cytologic smears of this region were sometimes obtained by introducing a small rough pad of compressed gauze through the mouth into thenasopharynxwith an upward-angled forceps [20]. Over the years, with the introduction in the clinical ENT practice of fiber optic scopes, recently implemented also with narrow-band imaging endoscopy (NBI), the endoscopic-assisted management of these lesions (together with imaging techniques) has become much easier [21, 22]. In the suspect of a malignancy, it is quite simple to perform an endoscopic endonasal biopsy in order to obtain an histologic diagnosis. With these implementations, cytologic smears and fine-needle aspiration cytology (FNAC) should not be considered as a diagnostic option for a nasopharyngeal lesion. Moreover, once the lesion pedicle was identified, endoscopic-assisted surgery permitted us a minimal invasive excisional biopsy. As also reported by Roh et al. [8], even in our experience we confirm that endoscopic surgery is effective to obtain the complete resection of benign nasopharyngeal tumors thus achieving surgical clear margins of resection with a low risk of injuries to the Eustachian tube.
